# Changes in fecal short‐chain fatty acids following fecal microbiota transplantation in patients with irritable bowel syndrome

**DOI:** 10.1111/nmo.13983

**Published:** 2020-09-17

**Authors:** Magdy El‐Salhy, Jørgen Valeur, Trygve Hausken, Jan Gunnar Hatlebakk

**Affiliations:** ^1^ Department of Medicine Stord Hospital Stord Norway; ^2^ Department of Clinical Medicine University of Bergen Bergen Norway; ^3^ Unger‐Vetlesen Institute Lovisenberg Diaconal Hospital Oslo Norway; ^4^ Department of Gastroenterology Oslo University Hospital Ullevål Oslo Norway; ^5^ Department of Medicine National Centre for Functional Gastrointestinal Disorders Haukeland University Hospital Bergen Norway

## Abstract

**Background:**

Short‐chain fatty acids (SCFAs) may play a role in the pathophysiology of irritable bowel syndrome (IBS). This study analyzed fecal SCFAs after performing fecal microbiota transplantation (FMT) in the IBS patients who were included in our previous study of the efficacy of FMT.

**Methods:**

This study included 142 of the 164 IBS patients who participated in our previous study. They were belonging to three groups: placebo (own feces), 30‐g (superdonor feces), and 60‐g (superdonor feces) FMT. The patients completed the IBS Severity Scoring System (IBS‐SSS) Birmingham IBS Symptom, Fatigue Assessment Scale (FAS), the IBS Quality of Life (IBS‐QoL) and Short‐Form Nepean Dyspepsia Index (SF‐NDI) questionnaires and delivered fecal samples at the baseline and 1 month after FMT. The SCFA levels were determined by vacuum distillation followed by gas chromatography.

**Key Results:**

The fecal butyric acid level was significantly increased after FMT in both the 30‐g and 60‐g groups (both *P* ≤ 0.001). In the 60‐g group, the levels of total SCFAs and isobutyric, isovaleric, and valeric acids increased after FMT. Butyric acid levels in the responders in both the 30‐g and 60‐g FMT groups were significantly inversely correlated with IBS‐SSS and FAS scores (*P* = 0.001, *r* = −0.3 and *P* = 0.0001. *r*=‐ 0.3, respectively). There were no differences in the SCFA levels in the placebo group after FMT.

**Conclusion and Inferences:**

FMT increases the fecal SCFA levels in IBS patients. The increase in the butyric acid level is inversely correlated with symptoms in IBS patients following FMT, suggesting that SCFAs might play a role in the pathophysiology of IBS. www.clini​caltr​ials.gov (NCT03822299).

**Key Points**

- Fecal short‐chain fatty acid (SCFA) levels differ between patients with irritable bowel syndrome (IBS) and healthy subjects. Abnormalities in SCFAs in patients with IBS might contribute to the pathophysiology of IBS.
- SCFAs increased after fecal microbiota transplantation (FMT) in patients with IBS. The compositions of fecal SCFAs at the baseline and after FMT differed with the IBS subtype. The changes in SCFAs varied with the applied FMT dose. The butyric acid level was inversely correlated with the total scores on the IBS Severity Scoring System and the Fatigue Assessment Scale.
- This study provides further evidence for the efficacy of FMT in treating IBS. This study suggests that intake of butyrate could be beneficial in the management of IBS.

## 1 INTRODUCTION

The intestinal bacterial profile in patients with irritable bowel syndrome (IBS) differs from that in healthy subjects.^2^ The intestine of IBS patients has a lower abundance of butyrate‐producing bacteria (Erysipelotrichaceae and Ruminococcaceae spp.) and a higher abundance of methane‐producing bacteria (Methanobacteriales spp.) compared with healthy subjects.^2^ In addition, the intestine of IBS patients has a higher abundance of *Lactobacillus* and *Ruminococcus* spp. and a lower abundance of *Bifidobacterium*,*Faecalibacterium,* and Erysipelotrichaceae spp. compared with healthy subjects.^2^ Moreover, the diversity of intestinal bacteria (dysbiosis) is lower in patients with IBS than in healthy subjects.^2^ In experimental animals, alterations in the intestinal bacteria are associated with gut dysmotility, visceral hypersensitivity, and altered intestinal permeability.^2^ All of these abnormalities are observed in patients with IBS.^2^ Thus, the gut microbiota is believed to play an important role in the pathophysiology of IBS.^2^ The gut microbiota is influenced by dietary modifications and nutritional supplements, which might explain the beneficial effects of different dietary regimes on IBS symptoms.^3^

Short‐chain fatty acids (SCFAs) are the main products of bacterial fermentation of undigested and unabsorbed carbohydrates in the intestine.^3^ SCFAs are the main energy source for colonic epithelial cells, regulating gut barrier functions and local immune defenses.^3^ Among the SCFAs, butyric acid has anti‐inflammatory and intestinal regenerative effects and administration of sodium butyrate to patients with inflammatory bowel disease changes the gut bacterial composition and induce the growth of butyric acid‐producing bacterial genera.^3^

The fecal SCFA levels differ between IBS patients and healthy subjects, with the fecal propionic acid level being significantly higher in IBS patients.^3^ Whereas the levels of propionic and butyric acids were lower in constipation‐predominant IBS (IBS‐C) patients than in healthy subjects, that of butyric acid in diarrhea‐predominant IBS (IBS‐D) patients was higher.^3^

A recent randomized double‐blind placebo‐controlled study performed by our group found that using a single superdonor for fecal microbiota transplantation (FMT) reduced IBS symptoms and fatigue and improved the quality of life in patients with IBS.^4^ These improvements were accompanied by marked changes in the bacterial profiles of the patients.^4^ The present study investigated whether FMT caused alterations in the fecal SCFAs in the same cohort of patients that we investigated in our previous study.

## 2 MATERIALS AND METHODS

### 2.1 Study design and randomization of patients

The design of this study has been described in detail previously.^4^ In brief, patients provided a fecal sample and completed five questionnaires to assess their symptoms and quality of life at the baseline and provided another fecal sample and completed a new set of questionnaires at 1 month after FMT. Polyethylene glycol and loperamide were allowed as rescue medication during the study. The patients were randomized to placebo (own feces), 30‐g (superdonor feces), or 60‐g (superdonor feces) FMT.^4^

### 2.2 Patients

This study included 142 of the 164 IBS patients who participated in our previous study.^4^ They were belonging to three groups: placebo (48 patients), 30‐g (50 patients), and 60‐g (44 patients) FMT. The characteristics of these patients are given in Table 1. The patients enrolled in this study have been described in detail previously.^4^ In brief, patients who fulfilled the Rome IV criteria for a diagnosis of IBS were recruited from those attending the outpatient clinic at Stord Hospital. None of the patients had previously consumed a low‐FODMAPs (fermentable oligo‐, di‐, monosaccharides, and polyols) diet, and all of the recruited patients had previously adhered to the National Institute for Health and Care Excellence (NICE)‐modified diet for at least 3 months without experiencing any marked improvement in symptoms; the patients were therefore considered as non‐responders to this diet.^4^ They also received a course of IBS treatment that slightly improved their symptoms.^4^ Dysbiosis in the fecal samples was done by the GA‐map Dysbiosis Test^®^ (Genetic Analysis) using 16S rRNA gene.^5^ The patients were not tested for small intestinal bacterial overgrowth or bile acid malabsorption.

**Table 1:** Baseline characteristics of patients

	Overall	Placebo	30‐g FMT	60‐g FMT	*p*
Number	142	48	50	44	
Age (y)	40.1 ± 13.1	41.4 ± 13.6	38.9 ± 12.4	38.9 ± 13.4	.665
Sex, female/male	118/24	42/6	38/12	38/6	.248
IBS‐D	52	16	20	16	
IBS‐C	54	21	17	16	.888
IBS‐M	36	11	13	12	
IBS duration (y)	15.5 ± 7.9	14.5 ± 8.0	17.2 ± 9.3	14.7 ± 5.7	.322
IBS‐SSS score	311.5 ± 77.0	310.5 ± 73.8	313.3 ± 76.7	310.6 ± 82.4	.989
Patients with MSS	59 (41.5%)	20 (41.7%)	21 (42%)	18 (40.9%)	.997
Patients with SSS	83 (58.5%)	28 (58.3%)	29 (58%)	26 (59.1%)	.998
FAS score	31.5 ± 5.0	30.6 ± 4.4	31.4 ± 5.3	31.4 ± 5.1	.976
Dysbiosis index	2.8 ± 1.1	2.7 ± 1.1	2.8 ± 1.0	2.9 ± 1.0	.781
Patients with dysbiosis	64%	67%	57%	67%	.578
PPI medication	55 (38.7%)	19 (39.6%)	18 (36.0%)	18 (40.9%)	.878
Birth control medication	84 (59.1%)	25 (52.1%)	29 (58.0%)	30 (68.2%)	.286
Antimigraine medication	10 (7.0%)	3 (6.2%)	4 (8.0%)	3 (6.3%)	.942
Medication against asthma/allergies	18 (12.7%)	6 (12.5%)	7 (14.0%)	5 (11.6%)	.928
Medication with levothyroxine	3 (2.1%)	1 (2.1%)	0 (0%)	2 (4.5%)	.311
Medication with heart/vascular drugs	6 (4.2%)	3 (6.3%)	2 (4.0%)	1 (2.3%)	.635

Note

Data are mean ± SD, *n*, %, or *n* (%) values.

PPI, proton‐pump inhibitor. MSS, moderate symptom severity (IBS‐SSS score between 175 and 300). SSS, severe symptom severity (IBS‐SSS score of ≥300).

The inclusion criteria were being aged 18‐85 years and having moderate‐to‐severe IBS symptoms, as indicated by a score of ≥175 on the IBS Severity Scoring System (IBS‐SSS). The exclusion criteria were the presence of systemic disease, immune deficiency or being treated by immune‐modulating medication, pregnant, planning pregnancy, lactating, having a severe psychiatric disorder, having alcohol or drug abuse, or taking probiotics, antibiotics, or IBS medications within 8 weeks prior to study inclusion.

### 2.3 Superdonor

The single superdonor used in this study has been described in detail previously. Briefly, he was screened according to the European guidelines for donors for FMT.^6^, ^7^ He was a healthy 36‐year‐old man, non‐smoker, not taking any medication regularly, and had a BMI of 23.5 kg/m^2^. He was born via a vaginal delivery, breastfed, and had taken a few courses of antibiotics during his life. He exercised regularly and took sport‐specific dietary supplements, which made his diet richer than average in protein, fiber, minerals, and vitamins.^4^

The GA‐map Dysbiosis Test^®^ revealed that he was normobiotic, but his fecal bacterial profile deviated from the expected normal abundance in 14 of the 39 bacteria markers examined: 12 bacteria in the Firmicutes phylum and 1 each in the Proteobacteria and Verrucomicrobia phyla.^4^ SCFAs were analyzed in donor's fecal samples taken at 0, 3, 6, 12, and 18 months.

### 2.4 Fecal sample collection, preparation, and administration

Fecal samples were frozen immediately and kept at −20°C until they were delivered frozen to the laboratory, where they were kept at −80°C. The process of FMT has been described in detail previously.^4^ In brief, the patients randomized to the placebo received 30 g of their own feces, while those in the 30‐g and 60‐g FMT groups received 30 and 60 g of the superdonor’s feces, respectively. The fecal material was thawed for 2 days at 4°C, mixed with 40 ml of sterile saline, and filtered before administration. The transplant was administered to the distal duodenum via the working channel of a gastroscope, followed by another 40 ml of sterile saline.

### 2.5 Symptom and quality‐of‐life assessments

Symptoms were assessed using the IBS‐SSS and the Birmingham IBS Symptom (Birmingham IBS‐S) questionnaires.^8^, ^9^ Fatigue was measured using the Fatigue Assessment Scale (FAS).^10^ Quality of life was measured using the IBS Quality of Life (IBS‐QoL) and Short‐Form Nepean Dyspepsia Index (SF‐NDI) questionnaires.^11^, ^12^, ^13^ Higher IBS‐QoL and lower SF‐NDI scores indicate a better quality of life. Patients who exhibited a decrease of ≥50 points in the total IBS‐SSS score after FMT were considered responders.

### 2.6 Determination of fecal SCFA levels

The fecal samples were weighed and homogenized with a solution containing 3 mmol/L 2‐ethylbutyric acid and 0.5 mmol/L H_2_SO_4_. A sample (2.5 mL) of the homogenate was vacuum‐distilled, and the SCFA levels were determined by gas chromatography (Agilent 7890 A; Agilent) using a capillary column (serial no. USE400345H; Agilent J&W GC columns, Agilent).^14^, ^15^ Flame ionization was used to determine the levels of total SCFAs, acetic, propionic, isobutyric, *n*‐butyric, isovaleric, *n*‐valeric acid, isocapronic, and *n*‐capronic acids, with the results expressed in units of mmol/kg wet weight.

### 2.7 Ethics

The study was approved by the Regional Committee for Medical and Health Research Ethics West, Bergen, Norway (approval no. 2017/1197/REK vest). All subjects provided both oral and written consents to participate. The study was registered at www.clini​caltr​ials.gov (NCT03822299) and www.crist​in.no (ID657402).

### 2.8 Statistical analysis

The sample size required in each arm of the study was 20 patients, as calculated by assuming that a placebo effect was 40% and an effect response was 80% (*α* = 0.05, 1−*β* = 0.80). We included 142 patients in order to avoid type 2 statistical errors. Differences between the placebo, 30, and 60‐g FMT groups in age, IBS‐SSS score, and FAS score were analyzed using one‐way ANOVA with Tukey’s multiple‐comparisons test as a post‐test. Differences between the baseline and after 1 month in Birmingham IBS‐S, IBS‐QoL, and SF‐NDI were analyzed using the Mann–Whitney test. Differences between the placebo, 30‐g, and 60‐g FMT groups in sex, overall responses, numbers of IBS subtypes included in the study, and IBS subtype medications were analyzed using the chi‐squared test. The paired *t* test was used to identify differences in the SCFA levels before and 1 month after FMT in the placebo, 30‐g, and 60‐g groups and with the IBS subtypes. Control for multiple testing with the SCFA parameters was not done. The correlations between the changes in SCFA levels and the IBS‐SSS and FAS scores were analyzed using the non‐parametric Spearman test. These analyses were performed using GraphPad Prism (version 8). All of the authors had access to the study data and reviewed and approved the final version of the manuscript.

## 3 RESULTS

### 3.1 Symptom assessment

**Figure 1:**
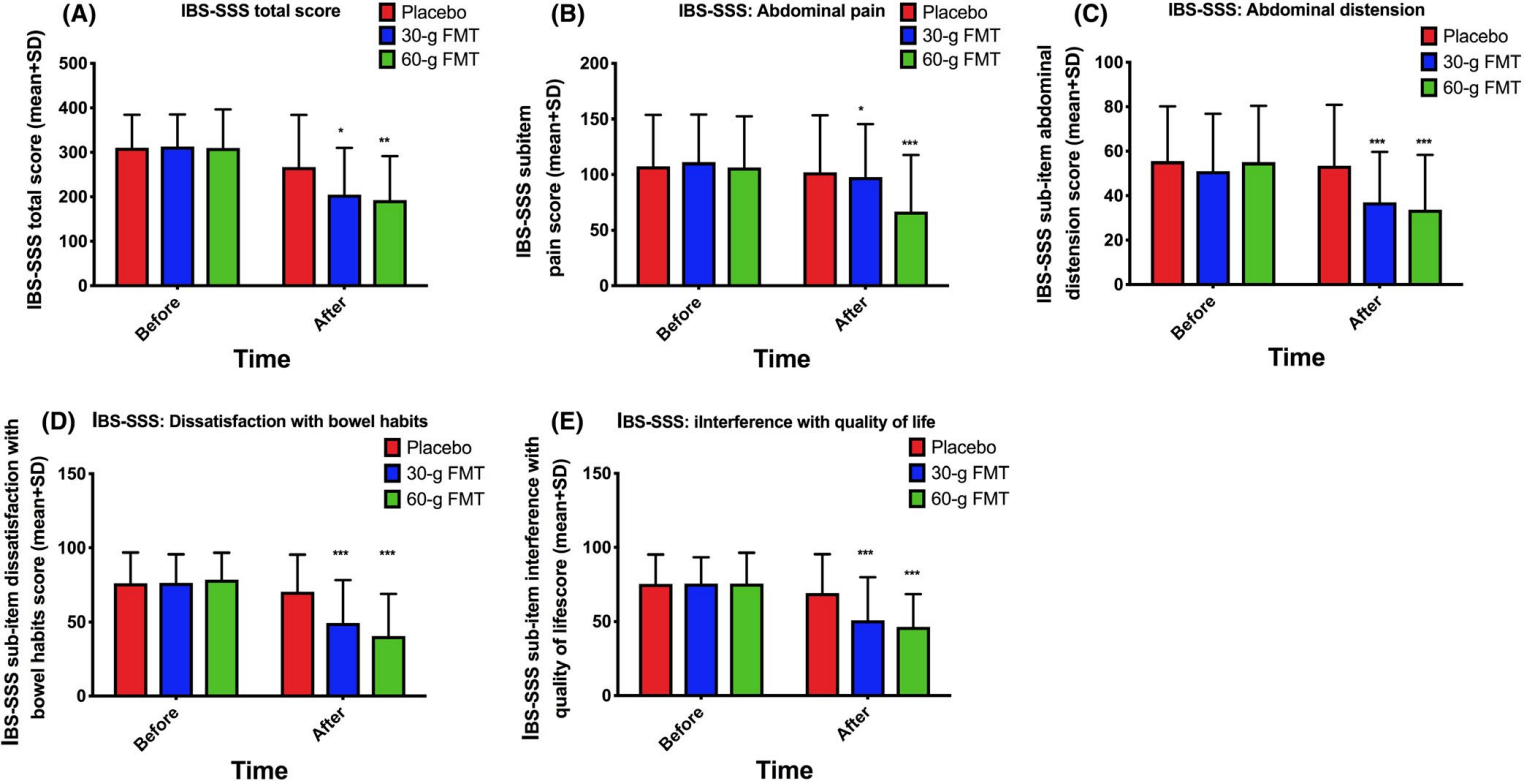
IBS‐SSS scores: total (A), abdominal pain (item 1) (B), abdominal distension (item 2) (C), dissatisfaction with bowel habits (item 3) (D), and interference with quality of life (item 4) (E). ^*^ *p* < 0.05; ^**^ *p* < 0.01; ^***^ *p* < 0.001 compared to placebo

**Figure 2:**
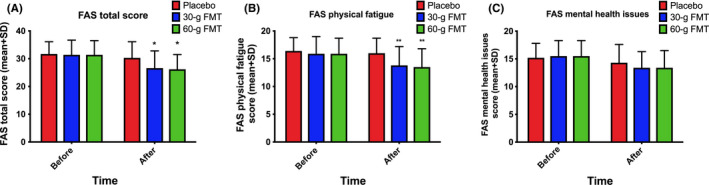
FAS scores: total (A), physical fatigue (B), and mental health (C). ^*^ *p *< 0.05; ^**^ *p* < 0.01 compared to placebo

The response rates to FMT were 18.2%, 74.0%, and 88.6% in the placebo, 30‐g, and 60‐g groups, respectively. The response differed significantly between the three groups (*P* < 0.0001) and between the 30‐g and 60‐g FMT groups (*P* = 0.01). The total and the sub‐items IBS‐SSS scores decreased significantly after FMT (Figure 1). Birmingham IBS‐S total score and its three domains were also significantly reduced after FMT (Table 2). The total FAS score was also significantly reduced after FMT. The scores for physical fatigue items were significantly reduced, while those for mental health issues were not (Figure 2). The total score of IBS‐QoL was significantly increased, and SF‐NDI total score was significantly decreased after FMT (Tables 3 and 4).

**Table 2:** The Birmingham IBS Severity Scoring System and its three domains in placebo and FMT‐treated patients

Time	Group	Total score (mean ± SD)	Pain (mean ± SD)	Diarrhea (mean ± SD)	Constipation (mean ± SD)
0	Placebo	23.2 ± 8.1	7.3 ± 3.3	7.8 ± 4.9	7.7 ± 3.7
	FMT 30 g	5.9 ± 5.7	8.3 ± 2.0	10.3 ± 4.2	7.3 ± 3.2
	FMT 60 g	25.7 ± 6.8	8.2 ± 2.7	9.2 ± 4.9	8.3 ± 3.2
1 month	Placebo	21.3 ± 8.2	7.2 ± 3.5	6.5 ± 4.9	7.5 ± 3.2
	FMT 30 g	19.0 ± 8.0****	6.0 ± 3.1***	6.9 ± 4.7***	5.7 ± 3.4*
	FMT 60 g	16.4 ± 8.6****	5.1 ± 3.1****	5.2 ± 4.0****	6.2 ± 4.9**

* *p* < 0.05;

** *p* < 0.01;

*** *p* 0.001;

**** P < 0.0001 as compared to baseline.

**Table 3:** IBS‐QoL total scores and scores in the eight domains of the scale in placebo and FMT‐treated patients

Time	Group	Total score	1	2	3	4	5	6	7	8
0	Placebo	115.2 ± 20.6	26.6 ± 6.4	22.9 ± 5.8	11.7 ± 2.8	10.5 ± 2.5	6.8 ± 2.8	14. ± 3.3	7.5 ± 1.9	11.1 ± 2.7
	30‐g FMT	109.8 ± 22.3	26.3 ± 6.8	19.8 ± 5.6	11.1 ± 3.4	10.4 ± 2.7	5.8 ± 2.7	13.7 ± 3.4	7.6 ± 1.9	10.9 ± 2.4
	60‐g FMT	113.1 ± 20.9	27.4 ± 6.5	21.0 ± 4.7	11.7 ± 2.6	10.6 ± 2.5	6.6 ± 2.8	14.0 ± 3.3	7.6 ± 1.7	10.9 ± 2.6
1 month	Placebo	121.9 ± 28.3	28.9 ± 7.4	23.0 ± 5.7	12.7 ± 4.5	12.2 ± 2.6^*^	8.0 ± 3.0	14.7 ± 3.7	7.3 ± 2.4	11.2 ± 3.1
	30‐g FMT	122.8 ± 23.3**	29.9 ± 6.8**	21.9 ± 4.8*	13.3 ± 3.8**	11.9 ± 2.3**	7.1 ± 2.9*	16.0 ± 3.0***	7.9 ± 1.7	11.6 ± 2.3
	60‐g FMT	126.1 ± 23.6**	30.9 ± 6.4**	22.7 ± 4.8*	13.8 ± 3.3**	12.0 ± 2.4*	8.4 ± 3.2**^*^	15.0 ± 3.7	8.2 ± 2.0	11.5 ± 2.5

Note

IBS‐QoL domains: 1, dysphoria; 2, interference with daily activities; 3, body image; 4, health worries; 5, food avoidance; 6, social reaction; 7, sexual function; and 8, impact on relations.

*p*<0.05;

*p* < 0.01;

*p* < 0.001.

**Table 4:** SF‐NDI total score and scores on the five subscales of this scale in placebo and FMT‐treated patients

Time	Group	Total score	1	2	3	4	5
0	Placebo	30.0 ± 7.6	6.4 ± 1.8	5.7 ± 1.9	6.8 ± 1.8	5.4 ± 2.1	5.4 ± 1.8
	30‐g FMT	30.3 ± 7.5	6.2 ± 1.6	5.8 ± 2.1	7.2 ± 2.1	5.4 ± 1.7	5.6 ± 2.3
	60‐g FMT	31.1 ± 8.2	6.6 ± 1.9	5.9 ± 2.3	7.1 ± 1.8	5.8 ± 1.9	5.7 ± 2.1
1 month	Placebo	28.5 ± 8.7	6.0 ± 2.1	5.4 ± 2.2	6.7 ± 2.2	4.9 ± 2.1	5.3 ± 2.2
	30‐g FMT	28.1.6 ± 8.1*	5.2 ± 1.9**	5.1 ± 2.1	6.5 ± 2.2	4.6 ± 1.7*	5.1 ± 2.2
	60‐g FMT	25.2 ± 9.9**	5.2 ± 2.1***	4.9 ± 2.4*	5.7 ± 2.3**	4.6 ± 2.0**	5.0 ± 2.7

Note

Data are mean ± SD values. SF‐NDI subscales: 1, tension; 2, interference with daily activities; 3, disruption to eating/drinking; 4, knowledge about/control over disease symptoms; and 5, interference with work/study.

*p* < 0.05;

*p* < 0.01;

Compared to baseline.

### 3.2 Fecal SCFA levels

The levels of total SCFAs and the individual SCFAs studied did not change after FMT in the placebo group (Table 5). The fecal butyric acid level increased significantly after FMT in the 30‐g group as a whole and in the responders, but not in the non‐responders (Table 6). In the 60‐g group, the fecal levels of total SCFAs, isobutyric acid, and butyric acid were higher after FMT in the whole group and in the responders, but not in the non‐responders. Moreover, the fecal levels of isovaleric and valeric acids increased in the responders after FMT (Table 7).

**Table 5:** SCFA levels in the feces of the placebo group before and after FMT

Acid	Total group			Responders			Non‐responders		
	Before FMT	After FMT	*p*	Before FMT	After FMT	*p*	Before FMT	After FMT	*p*
Total SCFAs	72.7 ± 36.7	68.2 ± 23.0	0.507	86.6 ± 36.0	83.6 ± 25.6	>0.999	72.7 ± 36.7	69.0 ± 23.2	0.914
Acetic acid	41.6 ± 17.5	40.4 ± 14.5	0.634	48.3 ± 19.4	47.7 ± 15.9	0.886	41.0 ± 17.4	39.7 ± 14.4	0.828
Propionic acid	12.1 ± 7.6	11.4 ± 5,0	0.515	15.6 ± 8.4	13.6 ± 5.4	>0.999	11.8 ± 7.6	11.2 ± 5.0	0.945
Isobutyric acid	1.5 ± 1.5	1.3 ± 0.8	0.466	1.8 ± 0.4	1.5 ± 1.0	0.800	1,4 ± 1.5	1.3 ± 0.8	0.485
Butyric acid	13.7 ± 8.8	12.2 ± 6.3	0.193	18.2 ± 13.9	16.6 ± 8.5	0.886	13.3 ± 8.3	11.8 ± 6.0	0.701
Isovaleric acid	2.2 ± 2.3	1.9 ± 1.3	0.372	2.6 ± 0.9	2.2 ± 1.7	0.914	2.2 ± 2.4	1.9 ± 1.3	0.738
Valeric acid	1.7 ± 1.5	1.4 ± 0.7	0.340	2.0 ± 0.7	1.8 ± 0.8	0.629	1.7 ± 1.5	1.4 ± 0.7	0.825
Isocapronic acid	0.10 ± 0.04	0.01 ± 0.07	0.860	0.40 ± 0.3	0.60 ± 0.9	0.771	0.50 ± 0.7	0.40 ± 0.7	0.768
Capronic acid	0.5 ± 0.7	0.5 ± 0.7	0.891	0.03 ± 0.05	0.0 ± 0.0	>0.999	0.01 ± 0.04	0.1 ± 0.08	0.936

Note

Data are mean ± SD values expressed in units of mmol/kg wet weight.

**Table 6:** Fecal SCFA levels in the 30‐g group before and after FMT

Acid	Total group			Responders			Non‐responders			Superdonor
	Before FMT	After FMT	*p*	Before FMT	After FMT	*p*	Before FMT	After FMT	*p*	
Total SCFAs	76.6 ± 39.6	69.2 ± 26.5	0.2	75.5 ± 42.1	68.6 ± 26.5	0.3	81.2 ± 28.0	71.6 ± 27.9	0.3	95.7 ± 21.8
Acetic acid	44.3 ± 21.4	40.2 ± 15.0	0.2	43.8 ± 23.0	39.8 ± 14.8	0.3	46.0 ± 14.1	41.5 ± 16.5	0.4	57.0 ± 10.0
Propionic acid	13.4 ± 9.5	11.4 ± 6.8	0.2	13.9 ± 10.2	11.8 ± 7.2	0.3	13.0 ± 6.1	11.5 ± 7.7	0.3	14.2 ± 3.9
Isobutyric acid	1.4 ± 0.9	1.3 ± 0.6	0.6	1.4 ± 1.0	1.4 ± 0.6	0.7	1.2 ± 0.7	1.2 ± 0.4	0.9	0.7
Butyric acid	10.6 ± 8.0	15.5 ± 10.2	0.005*	9.0 ± 6.2	15.8 ± 10.5	0.0007*	17.2 ± 10.9	13.5 ± 8.9	0.2	24.9 ± 6.7
Isovaleric acid	2.0 ± 1.4	1.9 ± 0.9	0.5	2.1 ± 1.4	1.9 ± 0.9	0.5	1.8 ± 1.0	1.8 ± 0.8	0.9	0.8 ± 0.2
Valeric acid	1.9 ± 1.9	1.6 ± 0.8	0.4	1.9 ± 2.1	1.6 ± 0.8	0.3	1.6 ± 0.9	1.7 ± 0.8	0.7	1.4 ± 0.5
Isocapronic acid	0.0 ± 0.0	0.0 ± 0.0	0.3	0.0 ± 0.0	0.0 ± 0.0	0.3	0.0 ± 0.0	0.0 ± 0.0	0.3	0.0 ± 0.0
Capronic acid	0.7 ± 1.3	0.5 ± 0.9	0.2	0.7 ± 1.3	0.5 ± 0.9	0.3	0.7 ± 1.0	0.4 ± 0.4	0.4	0.0 ± 0.0

Note

Data are mean ± SD values expressed in units of mmol/kg wet weight.

* Statistically significant.

**Table 7:** Fecal SCFA levels in the 60‐g group before and after FMT

Acid	Total group			Responders			Non‐responders			Superdonor
	Before FMT	After FMT	*p*	Before FMT	After FMT	*p*	Before FMT	After FMT	*p*	
Total SCFAs	71.6 ± 40.0	87.2	0.03*	74.5 ± 41.4	95.8 ± 47.9	0.009*	62.5 ± 29.2	62.6 ± 15.8	0.5	95.7 ± 21.8
Acetic acid	43.6 ± 19.7	46.1 ± 12.7	0.6	42.6 ± 17.6	45.8 ± 12.7	0.5	47.1 ± 38.1	53.3 ± 12.7	0.9	57.0 ± 10.0
Propionic acid	12.8 ± 8.4	13.6 ± 3.9	0.6	12.2 ± 6.6	13.6 ± 3.9	0.4	15.5 ± 15.1	13.5 ± 13.7	0.2	14.2 ± 3.9
Isobutyric acid	1.1 ± 0.6	1.9 ± 1.5	0.01*	1.2 ± 0.7	1.8 ± 1.0	0.003*	1.2 ± 0.6	2.0 ± 2.8	0.5	0.7 ± 0.1
Butyric acid	12.4 ± 8.9	17.8 ± 14.3	0.01*	11.9 ± 8.3	16.5 ± 10.9	**0.03** *	14.6 ± 11.8	24.0 ± 24.9	0.2	24.9 ± 6.7
Isovaleric acid	2.2 ± 2.5	2.4 ± 1.6	0.7	1.8 ± 1.0	2.6 ± 1.6	0.1*	3.8 ± 5.3	2.0 ± 1.5	0.3	0.8 ± 0.2
Valeric acid	1.8 ± 1.6	1.9 ± 1.2	0.6	1.5 ± 0.8	1.8 ± 1.1	0.04*	2.9 ± 3.2	2.1 ± 1.7	0.4	1.4 ± 0.5
Isocapronic acid	0.02 ± 0.08	0.01 ± 0.04	0.6	0.02 ± 0.08	0.01 ± 0.04	0.7	0.03 ± 0.05	0.01 ± 0.04	0.6	0.0 ± 0.0
Capronic acid	0.6 ± 0.8	0.6 ± 0.9	0.9	0.4 ± 0.6	0.4 ± 0.5	0.9	1.2 ± 1.1	1.2 ± 1.6	0.9	0.0 ± 0.0

Note

Data are mean ± SD values expressed in units of mmol/kg wet weight.

* Statistically significant.

At the baseline, the fecal butyric acid level was significantly higher in IBS‐D patients than in IBS‐C patients and patients with mixed‐diarrhea‐and‐constipation IBS (IBS‐M) (Table 8). The response to FMT did not differ significantly between the IBS subtypes in the placebo group regarding the levels of the total SCFAs or the other SCFA acids measured (Table 9). In the 30‐g group, the fecal butyric acid level increased after FMT in IBS‐C and IBS‐M patients, but not in IBS‐D patients. The response to FMT in terms of the changes in the levels of total SCFAs and other acids investigated did not differ between the IBS subtypes (Table 9).

**Table 8:** Differences between the IBS subgroups at the baseline

Acid	IBS‐D	IBS‐C	IBS‐M	*p*
Total SCFAs	72.8 ± 26.9	66.4 ± 26.4	86.3 ± 61.1	.412
Acetic acid	45.0 ± 16.7	38.5 ± 15.9	49.0 ± 27.8	238
Propionic acid	13.4 ± 7.7	11.6 ± 5.8	13.9 ± 13.3	.305
Isobutyric acid	1.3 ± 0.8	1.4 ± 0.8	1.8 ± 2.1	.676
Butyric acid	16.3 ± 9.9	10.7 ± 7.1^*^	10.0 ± 7.1^*^	**.009**
Isovaleric acid	1.9 ± 1.3	2.0 ± 1.2	3.0 ± 3.8	.725
Valeric acid	1.6 ± 0.8	1.8 ± 2.0	2.0 ± 2.2	.851
Isocapronic acid	0.017 ± 0.048	0.0 ± 0.0	0.04 ± 0.021	.155
Capronic acid	0.228 ± 0	0.400 ± 0.723	0.200 ± 0.520	.523

Note

Data are mean ± SD values expressed in units of mmol/kg wet weight.

* Statistically significant compared to IBS‐D.

**Table 9:** Fecal SCFA levels for the IBS subtypes in the placebo, 30‐g, and 60‐g groups before and after FMT

Acid	Group	IBS‐D		*p*	IBS‐C		*p*	IBS‐M		*p*
		Before	After		Before	After		Before	After	
Total SCFAs	Placebo	63.8 ± 22.2	66.5 ± 22.5	.742	71.3 ± 25.5	67.6 ± 24.0	.524	90.3 ± 64.0	75.9 ± 23.7	.508
	30‐g group	86.3 ± 26.5	80.2 ± 24.8	.388	66.8 ± 26.0	68.2 ± 25,9	.874	80.3 ± 62.3	59.0 ± 27.4	.306
	60‐g group	68.0 ± 24.9	88.8 ± 40.3	.016*	59.3 ± 27.6	92.5 ± 54.4	.011*	90.6 ± 62.6	93.6 ± 48.6	.811
Acetic acid	Placebo	37.7 ± 11.0	42.3 ± 15.6	.317	40.0 ± 16.0	38.4 ± 15.5	.627	49.1 ± 27.1	38.0 ± 10.0	.175
	30‐g group	53.0 ± 17.9	45.7 ± 14.7	.141	34.6 ± 11.4	37.8 ± 14.0	.392	44.9 ± 30.2	35.5 ± 15.4	.352
	60‐g group	38.0 ± 14.1	38.8 ± 14.7	.846	38.4 ± 17.3	48.4 ± 29.7	.209	56.4 ± 31.1	51.5 ± 24.7	.478
Propionic acid	Placebo	10.9 ± 4.7	10.8 ± 4.5	.935	10.5 ± 5.2	11.6 ± 5.4	.320	18.0 ± 13.1	12.2 ± 5.4	.220
	30‐g group	15.8 ± 9.6	13.9 ± 7.1	.502	11.7 ± 6.5	9.5 ± 5.5	.149	12.4 ± 12.8	10.2 ± 7.3	.645
	60‐g group	12.5 ± 6.8	12.9 ± 7.8	.740	13.7 ± 9.2	14.5 ± 11.1	.747	17.9 ± 13.1	15.0 ± 11.9	.303
Isobutyric acid	Placebo	1.3 ± 1.0	1.3 ± 1.0	.949	1.2 ± 9.5	1.2 ± 0.7	.906	2.3 ± 2.7	1.5 ± 0.6	.338
	30‐g group	1.3 ± 0.6	1.4 ± 0.6	.295	1.5 ± 1.1	1.4 ± 0.6	.623	1.5 ± 1.2	1.1 ± 0.3	.336
	60‐g group	1.3 ± 0.8	2.1 ± 1.3	.036*	1.2 ± 0.7	1.9 ± 1.6 ±	.029*	1.2 ± 0.3	1.2 ± 0.6	.855
Butyric acid	Placebo	12.9 ± 8.1	11.5 ± 5.6	.570	12.5 ± 8.2	11.9 ± 6.3	.726	19.0 ± 7.9	14.2 ± 7.9	.086
	30‐g group	16.2 ± 10.0	17.8 ± 8.3	.558	8.5 ± 5.0	15,4 ± 11.4	.007*	7.3 ± 4.8	18.5 ± 14.6	.023*
	60‐g group	12.5 ± 10.5	20.0 ± 15.9	.035*	13.2 ± 7.8	19.5 ± 14.7	.032*	11.4 ± 11.0	18.3 ± 15.2	.025*
Isovaleric acid	Placebo	2.0 ± 1.6	1.7 ± 1.5	.529	1.7 ± 0.8	1.9 ± 1.2	.547	3.4 ± 4.3	2.1 ± 1.1	.260
	30‐g group	1.8 ± 1.0	1.8 ± 1.1	.911	2.3 ± 1.6	1.9 ± 0.9	.437	2.1 ± 1.7	2.0 ± 0.6	.804
	60‐g group	1.9 ± 1.1	3.6 ± 2.1	.043*	1.8 ± 1.2	2.0 ± 1.2	.731	3.5 ± 4.8	2.5 ± 1.3	.474
Valeric acid	Placebo	1.5 ± 1.0	1.3 ± 0.8	.410	1.3 ± 0.6	1.5 ± 0.5	.356	2.7 ± 2.9	1.7 ± 0.6	.316
	30‐g group	1.6 ± 0.7	1.7 ± 0.7	.420	2.4 ± 3.0	1.5 ± 0.8	.207	1.5 ± 1.2	1.6 ± 0.8	.825
	60‐g group	1.5 ± 1.0	2.2 ± 1.7	.015*	1.6 ± 1.0	1.8 ± 1.2	.429	2.6 ± 2.9	1.9 ± 1.4	.315
Isocapronic acid	Placebo	0.013 ± 0.034	0.038 ± 0.126	.451	0.008 ± 040	0.000 ± 0.000	.328	0.012 ± 0.036	0.000 ± 0.000	.351
	30‐g group	0.0 ± 0.0	0.0 ± 0.0	.3	0.0 ± 0.0	0.0 ± 0.0	.3	0.0 ± 0.0	0.0 ± 0.0	.3
	60‐g group	0.008 ± 0.030	0.017 ± 0.058	.874	0.020 ± 0.082	0.013 ± 040	.555	0.0 ± 0.0	0.0 ± 0.001	.351
Capronic acid	Placebo	0.380 ± 0.694	0.380 ± 0.568	>.999	0.510 ± 0.735	0.546 ± 0.834	.900	0.482 ± 0.726	0.355 ± 0.836	.582
	30‐g group	0.7 ± 0.9	0.5 ± 0.8	.190	0.6 ± 1.0	0.5 ± 0.9	.428	1.1 ± 2.0	0.5 ± 0.7	.260
	60‐g group	1.4 ± 2.6	1.2 ± 2.1	.900	0.537 ± 0.747	0.640 ± 0.993	.434	0.689 ± 0.956	0.256 ± 0.381	.216

Note

Data are mean ± SD values expressed in units of mmol/kg wet weight. * Statistically significant compared to baseline.

In the 60‐g group, the response to FMT in terms of SCFA levels differed markedly between the IBS subtypes. The fecal butyric acid level increased for all of the IBS subtypes. The total fecal SCFA level increased in both IBS‐D and IBS‐C patients, but not in IBS‐M patients. The fecal levels of isovaleric and valeric acids increased in IBS‐D patients, but not in IBS‐C or IBS‐M patients (Table 9).

**Figure 3:**
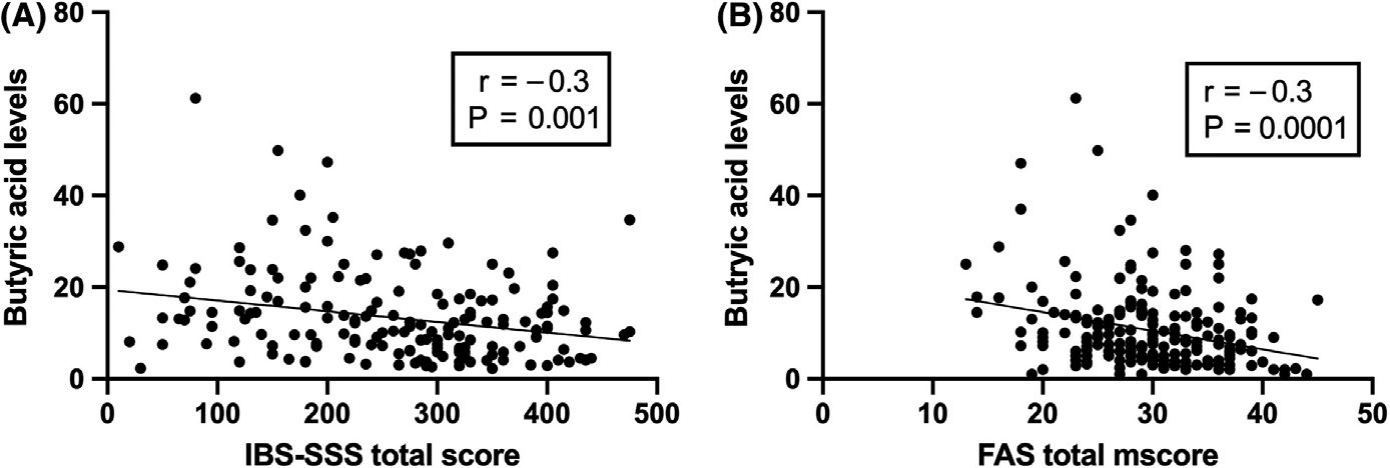
Correlation between butyric acid levels and IBS‐SSS total scores (A) and FAS total score (B)

The butyric acid level was inversely correlated with the IBS‐SSS and FAS scores (*p* = 0.005, *r* = −0.3, in both; Figure 3), while the SCFA level was inversely correlated with the IBS‐SSS score (*p* = 0.005, *r* = −0.4). Isobutyric acid, isovaleric acid, and valeric acids did not correlate with the IBS‐SSS total score (*p* = 0.2; *r* = −0.2, *p* = 0.2; *r* = 0.2, and *p* = 0.2; *r* = − 0.1, respectively). They did not correlate either with FAS total score (*p* = 0.6; *r* = 0.7, *p* = 0.5;*r* = 0.1, and *p* = 0.9; *r* = −0.2, respectively).

### 3.3 Adverse events

The adverse events were presented in detail previously, shortly about 20% of the patients treated with FMT experienced abdominal pain, cramping, tenderness, diarrhea, or constipation, compared with 2% of those in the placebo group. These adverse events were mild, self‐limiting, and occurred during the first 2 days after FMT.^4^

## 4 DISCUSSION

The present study showed that FMT‐induced marked changes in the fecal SCFAs levels in patients with IBS. These changes varied with the transplant dose, while the alterations in the SCFAs levels in response to FMT differed with the IBS subtype. In both the 30‐g and 60‐g groups, the fecal butyric acid level increased significantly in the responders after FMT. However, in the 60‐g group, the fecal levels of total SCFAs, isobutyric, isovaleric, and valeric acids increased in the responders following FMT.

At the baseline, the fecal butyric acid level was lower in IBS‐C and IBS‐M patients than in IBS‐D patients. This finding agrees with a previous report of the butyric acid level being lower in IBS‐C patients than in healthy subjects.^16^ The propionic acid level did not differ significantly between the IBS subtypes in the present study, which disagrees with the previous report of the propionic acid level being lower in IBS‐C patients than in healthy subjects.^16^ While the fecal butyric acid level increased in IBS‐C and IBS‐M patients after 30‐g FMT, that in IBS‐D patients did not change. The fecal butyric acid levels increased in all IBS subtypes following 60‐g FMT. The total fecal SCFA and isobutyric acid levels increased in both IBS‐D and IBS‐C patients, but not in IBS‐M patients. The fecal levels of isovaleric and valeric acids increased only in IBS‐D patients. The fermentation and production of SCFAs occur in the proximal colon and most of the SCFAs are absorbed rapidly by the colon epithelial cells, which means that the intestinal transit time affects the fecal SCFA levels.^17^ The differences in the fecal levels of different SCFAs between the IBS subtypes may therefore be caused by differences in the intestinal transit time between these subtypes. SCFAs regulate the secretion and absorption of water and electrolytes and intestinal motility.^18^, ^19^ SCFAs increase also the secretion and upregulate the gene expression of peptide YY,^20^, ^21^ which is a mediator of the ileal brake and stimulates the absorption of water and electrolytes in the large intestine.^18^, ^22^, ^23^ It is also possible that the differences in the SCFA levels between the IBS subtypes were due to differences in intestinal secretion, absorption, and motility.

In our previous study involving the same cohort of patients, the fecal levels of *Eubacterium* and *Lactobacillus* spp. were significantly increased after FMT.^4^ *Eubacterium* spp. are among the bacteria that produce butyrate upon carbohydrate fermentation, and treatment with *Lactobacillus* spp. can reportedly increase the fecal butyrate level.^24^, ^25^ These previously reported changes in the intestinal bacterial profile can explain the increase in the fecal butyric acid level in both the 30‐g and 60‐g groups after FMT observed in this study. Butyrate is an important source of energy for colonic epithelial cells, and its lack can cause mucosal atrophy.^19^ Butyrate also affects the immune response, modulates the oxidative stress of the host, and decreases intestinal cell permeability and intestinal motility.^22^ Furthermore, butyrate appears to modulate colonic hypersensitivity, and treatment with butyrate reduces the abdominal pain in patients with IBS.^26^, ^27^, ^28^ In the present study, the butyric acid level was inversely correlated with abdominal symptoms and fatigue, suggesting its role in the pathophysiology of IBS. The role of the increased levels of isobutyric, isovaleric, and valeric acids in the 60‐g group after FMT is not clear. Further studies using animal models may shed light on the pathophysiology of SCFAs in IBS.^29^

One of the main strengths of this study is that it investigated a relatively large cohort of patients with IBS, included three of the four IBS subtypes, and used a single superdonor. However, the study also had limitations: It did not include the unclassified IBS subtype (IBS‐U), it did not investigate the long‐term effects of FMT on fecal SCFA levels, and the cohort of included patients were those who did respond to the modified NICE diet; this means that the outcomes cannot be applied to the whole IBS population. Moreover, it is a single‐center study whose main findings are yet to be replicated in larger multicenter studies.

## CONFLICT OF INTEREST

The authors have nothing to disclose.

## AUTHOR CONTRIBUTIONS

M.E.S. designed the study; obtained the funding; administered the study; recruited the patients; performed FMT; collected, analyzed, and interpreted the data; and drafted the manuscript. J.V. contributed to the design of the study, analyzed the SCFAs, and critically revised the manuscript for important intellectual content. T.H. and J.G.H. contributed to the design of the study and to the analysis and interpretation of the data, and critically revised the manuscript for important intellectual content.

## References

[1] El‐Salhy M , Valeur J , Hausken T , Hatlebakk JG . Changes in fecal short‐chain fatty acids (SCFA) following fecal microbiota transplantation (FMT) in patients with irritable bowel syndrome (IBS). Neurogastroenterol Motil.2021;32(supplies. 1). 10.1111/nmo.13983 PMC7900992 32945066

[2] El‐Salhy M , Hatlebakk JG , Hausken T . Diet in irritable bowel syndrome (IBS): interaction with gut microbiota and gut hormones. Nutrients. 2019;11(8):1824. 10.3390/nu11081824 PMC6723613 31394793

[3] Facchin S , Vitulo N , Calgaro M , et al. Microbiota changes induced by microencapsulated sodium butyrate in patients with inflammatory bowel disease. Neurogastroenterol Motil. 2020:e13914. 10.1111/nmo.13914 PMC7583468 32476236

[4] El‐Salhy M , Hatlebakk JG , Gilja OH , Bråthen Kristoffersen A , Hausken T . Efficacy of faecal microbiota transplantation for patients with irritable bowel syndrome in a randomised, double‐blind, placebo‐controlled study. Gut. 2020;69(5):859–867. 31852769 10.1136/gutjnl-2019-319630 PMC7229896

[5] Casén C , Vebø HC , Sekelja M , et al. Deviations in human gut microbiota: a novel diagnostic test for determining dysbiosis in patients with IBS or IBD. Aliment Pharmacol Thera. 2015;42(1):71–83. 10.1111/apt.13236 PMC5029765 25973666

[6] El‐Salhy M , Mazzawi T . Fecal microbiota transplantation for managing irritable bowel syndrome. Expert rev Gastroenterol Hepatol. 2018;12(5):439–445. 29493330 10.1080/17474124.2018.1447380

[7] Cammarota G , Ianiro G , Tilg H , et al. European consensus conference on faecal microbiota transplantation in clinical practice. Gut. 2017;66:569–580. 28087657 10.1136/gutjnl-2016-313017 PMC5529972

[8] Francis CY , Morris J , Whorwell PJ . The irritable bowel severity scoring system: a simple method of monitoring irritable bowel syndrome and its progress. Aliment Pharmacol Thera. 1997;11(2):395–402. 10.1046/j.1365-2036.1997.142318000.x 9146781

[9] Roalfe AK , Roberts LM , Wilson S . Evaluation of the Birmingham IBS symptom questionnaire. BMC Gastroenterol. 2008;8:30. 18651941 10.1186/1471-230X-8-30 PMC2496907

[10] Hendriks C , Drent M , Elfferich M , De Vries J . The Fatigue Assessment Scale: quality and availability in sarcoidosis and other diseases. Curr Opin Pulm Med. 2018;24(5):495–503. 29889115 10.1097/MCP.0000000000000496

[11] Drossman DA , Patrick DL , Whitehead WE , et al. Further validation of the IBS‐QOL: a disease‐specific quality‐of-life questionnaire. Ame J Gastroenterol. 2000;95:999–1007. 10.1111/j.1572-0241.2000.01941.x 10763950

[12] Wong RK , Drossman DA . Quality of life measures in irritable bowel syndrome. Expert Rev Gastroenterol Hepatol. 2010;4(3):277–284. 20528115 10.1586/egh.10.19

[13] Arslan G , Lind R , Olafsson S , Florvaag E , Berstad A . Quality of life in patients with subjective food hypersensitivity: applicability of the 10‐item short form of the Nepean Dyspepsia Index. Dig Dis Sci. 2004;49:680–687. 15185878 10.1023/b:ddas.0000026318.81635.3b

[14] Zijlstra JB , Beukema J , Wolthers BG , Byrne BM , Groen A , Dankert J . Pretreatment methods prior to gas chromatographic analysis of volatile fatty acids from faecal samples. Clin Chimi Acta. 1977;78(2):243–250. 10.1016/0009-8981(77)90312-6 884859

[15] Hoverstad T , Fausa O , Bjorneklett A , Bohmer T . Short‐chain fatty acids in the normal human feces. Scand J Gastroenterol. 1984;19(3):375–381. 6740214

[16] Sun Q , Jia Q , Song L , Duan L . Alterations in fecal short‐chain fatty acids in patients with irritable bowel syndrome: A systematic review and meta‐analysis. Medicine. 2019;98(7):e14513. 10.1097/MD.0000000000014513 PMC6408019 30762787

[17] Lewis SJ , Heaton KW . Increasing butyrate concentration in the distal colon by accelerating intestinal transit. Gut. 1997;41(2):245–251. 9301506 10.1136/gut.41.2.245 PMC1891451

[18] Soret R , Chevalier J , De Coppet P , et al. Short‐chain fatty acids regulate the enteric neurons and control gastrointestinal motility in rats. Gastroenterology. 2010;138:1772–1782. 20152836 10.1053/j.gastro.2010.01.053

[19] Hamer HM , Jonkers D , Venema K , Vanhoutvin S , Troost FJ , Brummer RJ . Review article: the role of butyrate on colonic function. Aliment Pharmacol Ther. 2008;27:104–119. 17973645 10.1111/j.1365-2036.2007.03562.x

[20] Zhou J , Martin RJ , Tulley RT , et al. Dietary resistant starch upregulates total GLP‐1 and PYY in a sustained day‐long manner through fermentation in rodents. Am J Physiol Endocrinol Metab. 2008;295:E1160–E1166. 10.1152/ajpendo.90637.2008 PMC2584810 18796545

[21] Karaki S‐I , Mitsui R , Hayashi H , et al. Short‐chain fatty acid receptor, GPR43, is expressed by enteroendocrine cells and mucosal mast cells in rat intestine. Cell Tissue Res. 2006;324:353–360. 16453106 10.1007/s00441-005-0140-x

[22] El‐Salhy M , Hatlebakk JG , Hausken T . Possible role of peptide YY (PYY) in the pathophysiology of irritable bowel syndrome (IBS). Neuropeptides. 2020;79:101973. 31727345 10.1016/j.npep.2019.101973

[23] Hamer HM , Jonkers DMAE , Bast A , et al. Butyrate modulates oxidative stress in the colonic mucosa of healthy humans. Clin Nutr. 2009;28:88–93. 19108937 10.1016/j.clnu.2008.11.002

[24] Zaleski A , Banaszkiewicz A , Walkowiak J . Butyric acid in irritable bowel syndrome. Przeglad Gastroenterologiczny. 2013;8(6):350–353. 24868283 10.5114/pg.2013.39917 PMC4027835

[25] Cremon C , Guglielmetti S , Gargari G , et al. Effect of Lactobacillus paracasei CNCM I‐1572 on symptoms, gut microbiota, short chain fatty acids, and immune activation in patients with irritable bowel syndrome: a pilot randomized clinical trial. United Eur Gastroenterol J. 2018;6(4):604–613. 10.1177/2050640617736478 PMC5987284 29881616

[26] Zhang J , Song L , Wang Y , et al. Beneficial effect of butyrate‐producing Lachnospiraceae on stress‐induced visceral hypersensitivity in rats. J Gastroenterol Hepatol. 2019;34(8):1368–1376. 30402954 10.1111/jgh.14536 PMC7379616

[27] Long X , Li M , Li L‐X , et al. Butyrate promotes visceral hypersensitivity in an IBS‐like model via enteric glial cell‐derived nerve growth factor. Neurogastroenterol Motil. 2018;30(4):e13227. 10.1111/nmo.13227 29052293

[28] Banasiewicz T , Krokowicz Ł , Stojcev Z , et al. Microencapsulated sodium butyrate reduces the frequency of abdominal pain in patients with irritable bowel syndrome. Colorectal Dis. 2013;15(2):204–209. 22738315 10.1111/j.1463-1318.2012.03152.x

[29] Johnson AC , Farmer AD , Ness TJ , Greenwood‐Van MB . Critical evaluation of animal models of visceral pain for therapeutics development: A focus on irritable bowel syndrome. Neurogastroenterol Motil. 2020;32(4):e13776. 31833625 10.1111/nmo.13776 PMC7890461

